# Cost and disease burden of Dengue in Cambodia

**DOI:** 10.1186/1471-2458-10-521

**Published:** 2010-08-31

**Authors:** Julien Beauté, Sirenda Vong

**Affiliations:** 1 Epidemiology and Public Health Unit, Institut Pasteur in Cambodia, Bd Monivong, 5, BP 983 Phnom Penh, Cambodia

## Abstract

**Background:**

Dengue is endemic in Cambodia (pop. estimates 14.4 million), a country with poor health and economic indicators. Disease burden estimates help decision makers in setting priorities. Using recent estimates of dengue incidence in Cambodia, we estimated the cost of dengue and its burden using disability adjusted life years (DALYs).

**Methods:**

Recent population-based cohort data were used to calculate direct and productive costs, and DALYs. Health seeking behaviors were taken into account in cost estimates. Specific age group incidence estimates were used in DALYs calculation.

**Results:**

The mean cost per dengue case varied from US$36 - $75 over 2006-2008 respectively, resulting in an overall annual cost from US$3,327,284 in 2008 to US$14,429,513 during a large epidemic in 2007. Patients sustain the highest share of costs by paying an average of 78% of total costs and 63% of direct medical costs. DALY rates per 100,000 individuals ranged from 24.3 to 100.6 in 2007-2008 with 80% on average due to premature mortality.

**Conclusion:**

Our analysis confirmed the high societal and individual family burden of dengue. Total costs represented between 0.03 and 0.17% of Gross Domestic Product. Health seeking behavior has a major impact on costs. The more accurate estimate used in this study will better allow decision makers to account for dengue costs particularly among the poor when balancing the benefits of introducing a potentially effective dengue vaccine.

## Background

Dengue is the most important mosquito-borne viral disease worldwide. It is often endemic across tropical and subtropical areas [1]. Most forms of the disease are self-limited and vary from undifferentiated fever to classic and painful dengue fever (DF); however, on rare occurrence - particularly among children - complications occur in the forms of dengue hemorrhagic fever (DHF) or dengue shock syndrome (DSS) [1]. About 3.6 billion are at-risk of infection causing 500 million infections globally each year. Over nine million cases of DF and 18,000 deaths may occur annually [2].

In the context of endemicity, dengue is highly visible in the community, eliciting fear for dengue complications among mothers particularly during the dengue season. Although many developing countries recognize dengue as a public health concern, little funds are devoted to control measures. This neglect is likely related to a number of factors including the absence of specific treatment, the fact that the vaccine is still in development stage and that effective vector control interventions are too expensive for any developing countries. Above all, compared to other major infectious diseases, dengue ranks low internationally in terms of disability adjusted life years (DALYs) [3].

Evidence on the societal costs of the disease should help decision makers in setting priorities especially in the context of increasing health care costs and potential development of an effective vaccine. In this respect some Southeast Asian countries have produced cost of disease data at the regional and country level. Unfortunately these estimates are based on commonly underreported national surveillance data and failed to account for non-hospitalized cases or cases presenting with dengue related undifferentiated fever that occur in the community[4-7].

Dengue is endemic - affecting mainly children - in Cambodia (estimated population 14.4 M). Gross National Income per capita was US$600 in 2008 http://web.worldbank.org/. One international study included Cambodia but with scarce specific data [7]. The World health Organization (WHO) also estimated the burden of dengue in Cambodia but level of evidence remained low in absence of sound data [3]. In the light of recent estimates of true dengue disease incidence in Cambodia, this study aimed at estimating the accurate cost of dengue and its burden using DALYs. Public expenditure and also individuals' out-of-pocket payments were considered in the analysis. All calculations were performed for three years (2006, 2007 and 2008) in order to assess annual variations.

## Methods

### Estimates of dengue disease incidence

We used dengue incidence and case fatality rates as provided by Vong et al who conducted an active community-based surveillance (ACS) among individuals aged 0-19 years in Kampong Cham province, Cambodia from 2006 to 2008 [8]. Most of the population (~80%) lives in the southern and northwestern part of the country, mainly along the Mekong River. Kampong Cham is a central province and the most populated of Cambodia. Conditions of dengue's transmission are probably quiet representative of the whole country. A total of 42 villages were under active fever surveillance comprising a total study population of 14,354 participants. For groups of people aged above 20 years, it was assumed that incidences were constant. Since the vast majority of dengue cases in Cambodia occurred before age 20, assumptions made for adults appeared reasonable [9].

### Cost of illness

In a recent review, Tarricone *et al *suggested that cost of illness studies (COI) have to measure as accurately as possible "the true cost to society" to usefully inform decision makers. To achieve this goal, COI must include estimates of costs components and identification of subjects that bear the costs [10]. We used the methods of Tarricon*e et al *to estimate the cost of dengue in Cambodia. Medical resources include direct treatment costs (hospital costs, out patients costs, medication) and direct control costs (vector control programs, prophylaxis) [11]. Productive costs or indirect costs are costs related to morbidity and premature mortality [10,11]. We used disease burden incidence data calculated annually for the years 2006-8. To estimate costs, a bottom-up approach was chosen. We used dengue incidence and case fatality rates as provided by Vong et al. Results from the ACS suggested that health-seeking behavior changed from year to year. While 95% of dengue patients sought care in 2006, only about 50% and less than 40% sought care in 2007 and 2008 respectively [8]. Incidence rates and health seeking behaviors are displayed in table 1. For sensitivity analyses, we held health-seeking behaviors observed in 2006 for 2007 and 2008. We assumed that 2007 large epidemic could have had an impact on "usual" health care consumption. The share of private providers (private clinics and pharmacy) was consistent with the results of the Cambodian Health Survey [12]. Assumptions on costs distribution among government, families (Out-of-pocket payments) and other organizations (mainly Non Governmental Organizations (NGOs)) are displayed on Table 2. Public hospitals and health centers receive public subsidies that represent 50% of the total expenditure. In addition, another 20% of total costs are born by Health equity funds or other mechanisms of health insurance. Out-of-pocket payments account for the remaining 30%. Private clinics and pharmacies are fully financed by user fees. Costs figures were derived from specific studies carried out on Dengue and access to heath care in Cambodia [13-15]. It was assumed that only one provider delivered health care although qualitative studies suggest that several health services could be sought in some cases [16]. To estimate productive costs, a Human Capital Approach (HCA) was chosen in absence of sound data of the work market in Cambodia. The World Bank estimated average annual income of US$600. For each dengue fever episode, an average of 5 workdays lost was taken, which was consistent with previous studies [17]. It was assumed that this loss was equal for adults and children since at least one parent has to forgo his/her activity to care his/her sick child. In prevalence-based studies, lost expected earnings caused by premature mortality are assigned to the year of death [10]. Thus, future losses due to premature mortality were estimated using life expectancy at age of death with a starting age of work set at 15 years. A discount rate of 3% per year was applied in the baseline case and a flat rate was used for sensitivity analyses.

**Table 1:** Estimated number of total cases, hospitalized cases and fatal cases of dengue disease in Cambodia, incidence by group of age and health seeking behavior by year

	2006	2007	2008
Estimated No. cases	76,933	404,165	121,007
Estimated No. hospitalized cases	31,983	42,704	3,129
Estimated No. fatal cases	153	407	72
Incidence rate			
<5 years	16.0	79.2	15.5
5-9 years	15.8	83.7	24.4
10-14 years	8.9	46.2	17.2
15-19 years	1.1	15.2	6.8
>20 years	0.5*	1.5*	1.0*
Health seeking behavior			
% who seek care	93	50	37
% public hospital	42	11	3
% private clinic	48	31	28
% health center	2	4	1
% pharmacy	1	5	5

* Expert estimates

**Table 2:** Unit costs and their distribution

	Unit cost ($US)	Distribution (%)			Reference
		**Public**	**User fees**	**Other**	

Direct costs					
Direct medical costs					
Public hospital	67	50	30	20	(13-15)
Private clinic	20		100		
Health center	33	50	30	20	
Pharmacy	5		100		
Direct control costs					
Larviciding campaign	500,000	100			(32)
Productive costs					
Morbidity	8		100		World Bank
Mortality	600*		100		

*With an annual discount rate of 3%

### Burden of disease

DALYs were calculated using Murray's formula [18] but also taking into account recent remarks aiming at improving comparability and transferability of results [19]. Therefore, local life expectancy was used instead of standard life expectancy. Thus, DALYs are estimated by summing years of life lost (YLLs) and years of life lived with disability (YLDs).

$$YLLs\left[r,K,\beta \right]=\frac{KCe^{ra}}{{\left(r+\beta \right)}^{2}}\left\{e^{-\left(r+\beta \right)\left(L+a\right)}\left[-\left(r+\beta \right)\left(L+a\right)-1\right]-e^{-\left(r+\beta \right)a}\left[-(r+\beta )a-1\right]\right\}+\frac{1-K}{r}\left(1-e^{-rL}\right)$$

*Where*: *K *= age weighting factor; *C *= constant; *r *= discount rate; *a *= age of death; *β *= parameter from the age weighing function; *L *= expectation of life at age *a*.

$$YLDs\left[r,K,\beta \right]=D\{\frac{KCe^{ra}}{{\left(r+\beta \right)}^{2}}\left\{e^{-\left(r+\beta \right)\left(L+a\right)}\left[-\left(r+\beta \right)\left(L+a\right)-1\right]-e^{-\left(r+\beta \right)a}\left[-(r+\beta )a-1\right]\right\}+\frac{1-K}{r}\left(1-e^{-rL}\right)\}$$

We used the base case recommended by Murray and Lopez with *C *= 0.1658, *r *= 0.03, *K *= 1 and *β *= 0.04 [20]. Other factors were calculated for 17 groups of age in 5 year increments (<5 years, 5-9 years and so on until >80 years). Specific incidence rate of dengue, hospitalization and mortality were used for the first four groups of age (from <5 to 15-19 years) as provided by Vong et al [8]. As for previous similar studies carried out in other countries, a disability weight of 0.81 [4,21,22] and a duration of disability of 5 days were chosen [17].

## Results

### Cost of illness

**Figure 1:**
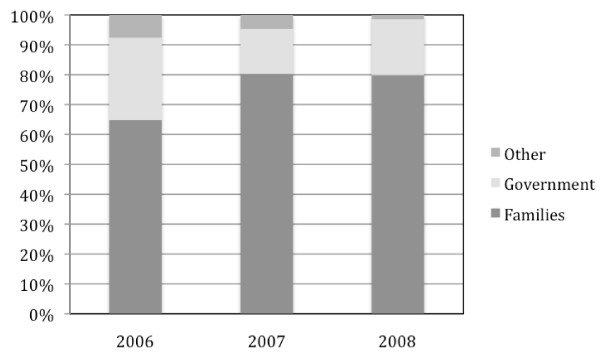
**Proportion of total costs born by families, government and other stakeholders**.

**Figure 2:**
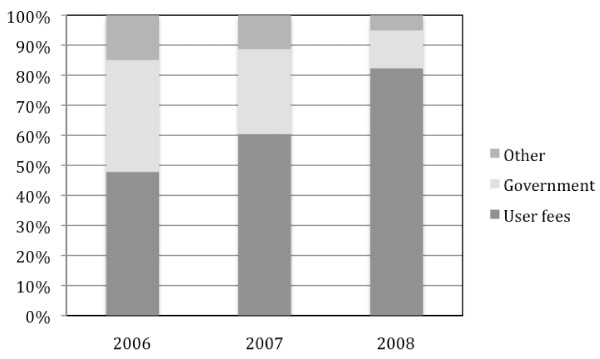
**Proportion of direct medical costs born by families, government and other stakeholders**.

Overall annual dengue costs ranged from US$3,327,284 in 2008 up to US$14,429,513 in 2007 with 2006 showing comparable amounts to 2008 (Table 3). In absence of a discount rate applied to future losses due to premature mortality, total costs doubled. On average the cost per dengue case was US$75 in 2006 and dropped to US$36 in 2007 and US$27 in 2008. Direct medical cost per case was $38 in 2006, $15 in 2007 and US$8 in 2008. When considering cases that sought care in both private and public sectors, these numbers rose to US$41 in 2006, US$29 in 2007 and US$21 in 2008 per case. Patients and their families support the highest share of costs with 65%, 80% and 80% of the total cost for 2006, 2007 and 2008 respectively (Fig. 1). When considering only direct medical costs, out-of-pocket payments represented 48%, 60% and 82% of the total for 2006, 2007 and 2008 respectively (Fig. 2). A sensitivity analysis was performed estimating costs in 2007 and 2008 with health seeking behaviors of 2006. Thus, costs in 2007 would be multiplied by 1.7 and costs of 2008 by 2.1.

**Table 3:** Costs by year ($US)

	2006	2007	2008
Total costs	5,771,079	14,429,513	3,327,284
Direct costs	3,437,570	6,432,840	1,463,189
Medical costs	2,937,570	5,932,840	963,189
Public hospitals	2,132,224	2,846,950	208,633
Private clinics	743,397	2,470,746	688,489
Health centers	57,628	508,384	34,772
Pharmacies	4,322	106,761	31,295
Control costs	500,000	500,000	500,000
Productive costs	2,333,509	7,996,673	1,864,095
Morbidity	632,325	3,321,906	994,579
Mortality (3% discount rate)	1,701,183	4,674,767	869,516
(Mortality (no discount))	(4,592,640)	(12,354,240)	(2,197,920)

### Burden of disease

Estimated DALY rates per 100,000 inhabitants ranged from 24.3 in 2008 to 116.6 in 2007 with 2006 in between at 40.0 (Table 4). The share due to premature mortality (Years of life lost/total DALYs) was 91%, 83% and 70% for 2006, 2007 and 2008 respectively.

**Table 4:** Estimated DALYs by year

	2006	2007	2008
Estimated total DALYs	5,603	16,330	3,397
Estimated YLLs	5,101	13,583	2,391
Estimated YDLs	502	2,747	1,006
Estimated DALYs rate/10^5^inhab.	40.0	116.6	24.3

DALYs: diability adjusted life years

YLLs: years of life lost

YDLs: years of life lived with disability

## Discussion

### On costs

Our analyses confirmed the high societal burden of dengue in Cambodia whose total costs represented between 0.03 and 0.17% of Cambodia's Gross Domestic Product. We feel that this analysis is a more accurate estimate of Cambodia's cost of illness due to dengue fever. These costs calculations were based upon robust estimates provided by the ACS of a large population. Unlike previous estimates, proportions of both hospitalized and ambulatory cases were accounted for [7]. Moreover, the study provided a longitudinal perspective of the burden of dengue through estimates from three years with differing epidemiological patterns. Indeed, the incidence data encompassed a regular seasonal epidemic year (2006), a large scale epidemic year (2007) and Year 2008 - a year following a major epidemic. This situation has been observed to be associated with a year of milder clinical presentation and lower incidence of dengue fever [23].

Nevertheless, these numbers still probably represent an underestimation of the real costs caused by endemic dengue. Mothers are well aware of the risks of potential severity during the dengue season. There is a local name for dengue "krun chheam" which translates into "hemorrhagic fever" in Khmer language. As such, during the rainy season fearing hemorrhagic dengue, it is thought that more mothers would seek hospital admission when their children present with febrile illnesses than that they would during the low dengue season. This phenomenon is likely to saturate hospitals capacities and to lower global health care quality because of an overwhelmed staff coping with commonly ill-equipped facilities. As defined by health economists, opportunity costs would occur when treatments for patients suffering from other diseases have to be postponed because of the dengue outbreak [11]. Our results underlined the importance of health seeking behavior on costs. The sensitivity analysis showed that changes in heath seeking behavior in 2007 decreased the medical costs.

COI aim at assessing the economic burden of a given disease to society. Since introduction of these studies, their utility in the decision-making process has often been disputed [10]. Some authors argue that COI merely identify diseases for which large amount of resources are already allocated [24]. COI could also fail in identifying diseases amenable by medical treatment or prevention [25]. Finally it is difficult to compare COI between countries of different health care capacity and access of individuals to adequate health care. Although incidence of dengue may be among the highest, COI may be proportionately lower compared with more developed neighboring countries as ill-equipped public and private hospitals would spend less on symptomatic treatment of dengue. Indeed, our results indicated that small amounts were devoted to dengue in Cambodia compared to other countries with an average cost per case around US$50 [7]. More importantly, despites recent effort by the Cambodian government to introduce safety nets (insurance) or health equity funds in hospitals, the burden of this cost is disproportionately born by the patients and their relatives. In 1997, Cambodia introduced user fees in order to increase hospital revenue, to limit under-the-table payments and eventually improve the quality of care [26,27]. It was thought that the system could improve financial sustainability and increase heath services utilization, as a consequence of higher quality health services [28]. Hitherto, results from early studies suggest that overall health care utilization increased following user fees introduction [29]. However, utilization of the poorer members of the community is not well documented. In addition nearly half of district hospitals were not covered by equity funds in 2006 [26]. Fee-exemption rates in Cambodia are known to be relatively low compared to other developing countries [26]. Since a significant proportion of Cambodians have no access or cannot afford private health care, many families forgo healthcare. This would be supported by recent findings that suggested a lower hospitalization rate for dengue in Cambodia's poorest households [14]. For the poorest, healthcare access or hospital costs remained an insurmountable issue [24] because loans and high interest rates can impoverish them for many years [14].

### On DALYs

Our results are consistent with previous studies. Sheppard *et al. *reported a DALY rate of 42 DALYs/100,000 in South East Asia [17] and the WHO estimated 54 DALYs/100,000 caused by dengue in Cambodia in 2004. However, Sheppard *et al. *found that 52% of DALYs were due to premature mortality whereas our results showed a higher share. Cambodia has a very young population with about 50% of the population under 20 years. Since duration of disability is very short and the vast majority of deaths occurred in the 0-20 age group, our estimate is probably more accurate. Some authors reproach age weighting in DALYs calculation to give more value to young adults than to other groups of age [30]. In the Cambodian demographic context, such assumption tends to underestimate the true burden of dengue. Compared to other DALYs estimates provided by the WHO, the burden of dengue appears similar to those of Japanese encephalitis (54 DALYs/100,000), trachoma (69 DALYs/100,000) or even malaria (143 DALYs/100,000). Regional comparisons suggest a burden equivalent in Thailand (60 DALYs/100,000) and Laos (93 DALYs/100,000).

### Limitations

As mentioned previously, opportunity costs were not taken into account and are likely to have an important impact on overall costs. Unfortunately, such data were not available and further studies are needed to document these costs. Incidence rates for groups of age above 20 years old were based on assumptions. Average income estimates were based on macroeconomic indicators since accurate data on both salaries and unemployment rate were not available. Median income is likely to be inferior to mean income in Cambodia because of a minority of people with very high incomes. Moreover, all estimates are subject to inaccuracy since informal work represents the majority of employment in Cambodia [31]. However, conservative values were taken on purpose to avoid any overestimation (1 dengue case per 1000 pop. on average).

### Implications for health policy

The question is to determine whether COI and DALYs are helpful in prioritizing resources allocation. At first glance, burden of dengue could appear as an issue little importance in Cambodia. DALYs caused by tuberculosis (1,712 DALYs/100,000) or meningitis (356 DALYs/100,000) are by far more considerable (WHO 2004 estimates). However, dengue strikes primarily children in Cambodia, resulting in a lesser impact on DALYs calculation. Regardless of costs underestimation, the share born by families clearly gives an indication of the true burden of dengue for Cambodians. Should a dengue vaccine be highly cost effective, its introduction in Cambodia would provide great benefits to the population [17] in terms of reduced incurrence of debt [14].

## Conclusions

To conclude, decision makers should take into account dengue costs particularly among the poor and the psychological stress caused by the disease in the population (assessed by their willingness to pay) when balancing the benefits of introducing a potentially effective dengue vaccine. In Cambodia, dengue is a health issue as much as an equity concern. This report establishes a methodological approach that uses more accurate estimates of dengue disease burden to suggest the importance of using cost indicators when considering the introduction of a dengue vaccine in Cambodia. Specific studies should also document the efficiency of other interventions. Previous studies performed in Cambodia reported contradictive results [9,32].

## Competing interests

None of the authors has any conflict of interest with the subject matter or materials discussed in the manuscript.

## Authors' contributions

JB and SV conceived of the study. SV compiled the secondary data. JB did the calculations. JB wrote the first draft. SV revised the manuscript. Both authors read and approved the final manuscript.

## Pre-publication history

The pre-publication history for this paper can be accessed here:

http://www.biomedcentral.com/1471-2458/10/521/prepub

## References

[B1] Halstead SB Dengue 2008 Imperial College Press

[B2] The global burden of disease 2004 update 2008 World Health Organization

[B3] Lopez AD Project DCP Global burden of disease and risk factors 2006 World Bank Publications

[B4] Anderson K Chunsuttiwat S Nisalak A Mammen M Libraty D Rothman A Burden of symptomatic dengue infection in children at primary school in Thailand: a prospective study Lancet 2007 369 9571 1452 9 10.1016/S0140-6736(07)60671-0 17467515

[B5] Clark D Mammen M Nisalak A Puthimethee V Endy T Economic impact of dengue fever/dengue hemorrhagic fever in Thailand at the family and population levels Am J Trop Med Hyg 2005 72 6 786 91 15964964

[B6] Garg P Nagpal J Khairnar P Seneviratne S Economic burden of dengue infections in India Trans R Soc Trop Med Hyg 2008 102 6 570 7 10.1016/j.trstmh.2008.02.015 18402995

[B7] Suaya J Shepard D Siqueira J Martelli C Lum L Tan L Cost of dengue cases in eight countries in the Americas and Asia: a prospective study Am J Trop Med Hyg 2009 80 5 846 55 19407136

[B8] Vong S Khieu V Glass O Ly S Ong S Huy R Dengue Incidence and Serotype-distribution in Semi-urban and Rural Cambodia: Results from Population-based Active Fever Surveillance, 2006-2008 PLoS Negl Trop Dis 2010 10.1371/journal.pntd.0000903 PMC2994922 21152061

[B9] Huy R Buchy P Conan A Ngan C Ong S Ali R National dengue surveillance in Cambodia 1980-2008: epidemiological and virological trends and the impact of vector control Bull World Health Organ 2010 10.2471/BLT.09.073908 PMC2930366 20865069

[B10] Tarricone R Cost-of-illness analysis. What room in health economics? Health Policy 2006 77 1 51 63 10.1016/j.healthpol.2005.07.016 16139925

[B11] Luyten J Beutels P Costing infectious disease outbreaks for economic evaluation: a review for hepatitis A Pharmacoeconomics 2009 27 5 379 89 10.2165/00019053-200927050-00003 19586076

[B12] Cambodia. National Institute of Statistics.; Cambodja. National Institute of Public Health Cambodia demographic and health survey 2005 2006 Calverton Md.: ORC Macro

[B13] Annear P Study of financial access to health services for the poor in Cambodia:phase 1:scope, design and data analysis 2006 Phnom Penh: P Annear

[B14] Huy R Wichmann O Beatty M Ngan C Duong S Margolis H Cost of dengue and other febrile illnesses to households in rural Cambodia: a prospective community-based case-control study BMC Public Health 2009 9 155 10.1186/1471-2458-9-155 19473500 PMC2696434

[B15] Van Damme W Van Leemput L Por I Hardeman W Meessen B Out-of-pocket health expenditure and debt in poor households: evidence from Cambodia Trop Med Int Health 2004 9 2 273 80 10.1046/j.1365-3156.2003.01194.x 15040566

[B16] Khun S Manderson L Health seeking and access to care for children with suspected dengue in Cambodia: an ethnographic study BMC Public Health 2007 7 262 10.1186/1471-2458-7-262 17892564 PMC2164964

[B17] Shepard D Suaya J Halstead S Nathan M Gubler D Mahoney R Cost-effectiveness of a pediatric dengue vaccine Vaccine 2004 22 9-10 1275 80 10.1016/j.vaccine.2003.09.019 15003657

[B18] Murray CJ Quantifying the burden of disease: the technical basis for disability-adjusted life years Bull World Health Organ 1994 72 3 429 445 8062401 PMC2486718

[B19] Fox-Rushby J Hanson K Calculating and presenting disability adjusted life years (DALYs) in cost-effectiveness analysis Health Policy Plan 2001 16 3 326 31 10.1093/heapol/16.3.326 11527874

[B20] Murray CJL Lopez AD Global Health Statistics: A Compendium of Incidence, Prevalence and Mortality Estimates for Over 200 Conditions 1996 1 Harvard School of Public Health

[B21] Luz P Grinsztejn B Galvani A Disability adjusted life years lost to dengue in Brazil Trop Med Int Health 2009 14 2 237 46 10.1111/j.1365-3156.2008.02203.x 19171013

[B22] Meltzer M Rigau-Perez J Clark G Reiter P Gubler D Using disability-adjusted life years to assess the economic impact of dengue in Puerto Rico: 1984-1994 Am J Trop Med Hyg 1998 59 2 265 71 9715944 10.4269/ajtmh.1998.59.265

[B23] Nisalak A Endy TP Nimmannitya S Kalayanarooj S Thisayakorn U Scott RM Serotype-specific dengue virus circulation and dengue disease in Bangkok, Thailand from 1973 to 1999 Am J Trop Med Hyg 2003 68 2 191 202 12641411

[B24] Shiell A Gerard K Donaldson C Cost of illness studies: An aid to decision-making? Health Policy 1987 8 3 317 323 10.1016/0168-8510(87)90007-8

[B25] Byford S Torgerson DJ Raftery J Cost of illness studies BMJ 2000 320 7245 1335 1335 10.1136/bmj.320.7245.1335 10807635 PMC1127320

[B26] Khun S Manderson L Poverty, user fees and ability to pay for health care for children with suspected dengue in rural Cambodia Int J Equity Health 2008 7 10 10.1186/1475-9276-7-10 18439268 PMC2386469

[B27] Akashi H Yamada T Huot E Kanal K Sugimoto T User fees at a public hospital in Cambodia: effects on hospital performance and provider attitudes Soc Sci Med 2004 58 3 553 64 10.1016/S0277-9536(03)00240-5 14652051

[B28] Gilson L The lessons of user fee experience in Africa Health Policy Plan 1997 12 4 273 285 10176263 10.1093/oxfordjournals.heapol.a018882

[B29] James CD Hanson K McPake B Balabanova D Gwatkin D Hopwood I To retain or remove user fees?: reflections on the current debate in low- and middle-income countries Appl Health Econ Health Policy 2006 5 3 137 153 10.2165/00148365-200605030-00001 17132029

[B30] Williams A Calculating the global burden of disease: time for a strategic reappraisal? Health Econ 1999 8 1 1 8 10.1002/(SICI)1099-1050(199902)8:1<1::AID-HEC399>3.0.CO;2-B 10082139

[B31] Informal economy, poverty and employment in Cambodia, Mongolia, Thailand: good practices and lessons learned: final report [Internet] 2007 http://www.ilo.org/asia/whatwedo/publications/lang--en/docName--WCMS_BK_PB_140_EN/index.htm [cited 2010 Apr 2]

[B32] Suaya J Shepard D Chang M Caram M Hoyer S Socheat D Cost-effectiveness of annual targeted larviciding campaigns in Cambodia against the dengue vector Aedes aegypti Trop Med Int Health 2007 12 9 1026 36 10.1111/j.1365-3156.2007.01889.x 17875014

